# Evaluation of models for predicting the probability of malignancy in patients with pulmonary nodules

**DOI:** 10.1042/BSR20193875

**Published:** 2020-02-28

**Authors:** You Li, Hui Hu, Ziwei Wu, Ge Yan, Tangwei Wu, Shuiyi Liu, Weiqun Chen, Zhongxin Lu

**Affiliations:** 1 Department of Medical Laboratory, the Central Hospital of Wuhan, Tongji Medical College, Huazhong University of Science and Technology, Wuhan 430014, China; 2 School of Laboratory Medicine, Hubei University of Traditional Chinese Medicine, Wuhan 430065, China; 3 Cancer Research Institute of Wuhan, The Central Hospital of Wuhan, Tongji Medical College, Huazhong University of Science and Technology, Wuhan 430014, China

**Keywords:** AUC, evaluation, lung cancer, prediction model, pulmonary nodule

## Abstract

**Objectives:** The post-imaging, mathematical predictive model was established by combining demographic and imaging characteristics with a pulmonary nodule risk score. The prediction model provides directions for the treatment of pulmonary nodules. Many studies have established predictive models for pulmonary nodules in different populations. However, the predictive factors contained in each model were significantly different. We hypothesized that applying different models to local research groups will make a difference in predicting the benign and malignant lung nodules, distinguishing between early and late lung cancers, and between adenocarcinoma and squamous cell carcinoma. In the present study, we compared four widely used and well-known mathematical prediction models.

**Materials and methods:** We performed a retrospective study of 496 patients from January 2017 to October 2019, they were diagnosed with nodules by pathological. We evaluate models’ performance by viewing 425 malignant and 71 benign patients’ computed tomography results. At the same time, we use the calibration curve and the area under the receiver operating characteristic curve whose abbreviation is AUC to assess one model’s predictive performance.

**Results:** We find that in distinguishing the Benign and the Malignancy, Peking University People’s Hospital model possessed excellent performance (AUC = 0.63), as well as differentiating between early and late lung cancers (AUC = 0.67) and identifying lung adenocarcinoma (AUC = 0.61). While in the identification of lung squamous cell carcinoma, the Veterans Affairs model performed the best (AUC = 0.69).

**Conclusions:** Geographic disparities are an extremely important influence factors, and which clinical features contained in the mathematical prediction model are the key to affect the precision and accuracy.

## Introduction

Pulmonary nodules are common. Referable to the characteristics of pulmonary nodules, computed tomography (CT) imaging is currently the most prior method for decreasing pulmonary nodules and screening early-stage lung cancer in high-risk populations [1]. The pulmonary nodule can be separated into solid nodules and subsolid nodules, usually, we divide subsolid nodules into pure ground glass nodules and partial solid nodules. At the same time, if a nodule completely masks the entire lung parenchyma, we can mention it as the solid nodule [2]. According to the size of the node, the pulmonary nodules with ≤8 mm are defined as subcentimeter nodules. The lesion with straight diameter >3 cm is defined as lung swelling (lung mass) rather than nodule. Based on previous research, the lung swelling with diameter >3 cm is usually malignant [3]. Consulting to the latest statistical data, among all diagnosed cancers, lung cancer occupies the first place account for 11.6% of the total number of cases, which constitutes 18.4% of total cancer deaths becomes the chief reason for cancer death; however, the different conditions of cancer in individual countries and regions indicate that significant geographical differences still exist [4]. Globally, countries that started to smoke early possessed high rate of smoking in the past, such as North America, Europe and so on. Interestingly, at present the number of smokers is decreasing in most of the countries (such as Australia, United Kingdom and United States). Unfortunately, smoking rates are rising in countries which began to smoke lately, especially for men. Above 50% of lung cancer patients died were from low and middle income countries every year [5]. Thanks to the widely used of CT scanning, there is a growing increase in the number of discovered pulmonary nodules, to a certain extent, it increases the survival rate for lung patients that achieves early detection and treatment Strategy. The National Lung Cancer Screening Test (NLST) revealed that via low-dose CT screening, we could roughly reduce 20% mortality of lung cancer as we compared it with chest radiography [6], which mainly attributed to technological developments in CT scanners and the employment of mainframe computer displays to display CT images.

There are two theories driving the purposes of lung nodule management. First, most lung nodules that are accidentally discovered or screen-detected are benign. Second, the overall 5-year survival rate for all lung cancer patients is approximately 18%, in which stage I account for 73–90% [7]. If CT finds that nodule density is benign calcification, intranodular fat-like low density (such as hamartoma) or arteriovenous malformations, follow-up observation or no follow-up can be made to avoid unnecessary examination and reduce the economic burden of patients. Accelerating-malignant nodules’ diagnosis and treatment and minimizing the detection of benign nodules are the concerns of all. For patients, there are so many factors that affect the development of malignant tumors, including age, gender and smoking history, as well as nodule size, location, shape, morphology, multiplicity and the presence of potential emphysema or fibrosis. The relative utility of each trait in predicting cancer possibilities has been extensively studied, but no single trait or combination has proven to be a reliable standard in this regard, this is partly due to the lack of any consistent or repeatable method to quantify gross morphological features as well as a lack of data regarding the clinical significance of the more subtle features [8]. Professional organizations have developed management guidelines for both screening and incidentally encountered nodules that are based on nodule size, morphology, and individual risk factors, and recommended intervals of computed tomographic (CT) follow-up to detect growth [9]. Since early treatment is so important, the establishment of predictive models is very necessary. The most widely verified models was Mayo Clinic Model. Veterans Affairs (VA) model, PKUPH Model, Brock Model. However, these models don’t only have various limitations, but also have different predictive parameters for each model, which has caused many problems for patients and clinicians. For example, the Mayo model does not suitable for patients diagnosed with cancer in the past 5 years or patients with the history of lung cancer [10]. Additionally, VA model does not apply to patients whose nodules smaller than 7 mm [11]. The PKUPH Model excludes patients with intrapulmonary and extrapulmonary malignancies within 5 years [12]. The Brock Model is suitable neither for screening low-risk populations nor for patients with hilar or mediastinal lymphadenopathy [13].

Model for assessing the probability of malignancy is a model for estimating malignant pulmonary nodules that was established by combining the current independent predictors of lung cancer and imaging features, and using statistical knowledge.

The research design of the model is the most important factor affecting the accuracy of this model. It is always a complicated problem to incorporate the characteristics of the patient into the prediction model. If a model can achieve such an effect that observed results are consistent with the predicted results, as well as can distinguish between high-risk and low-risk groups, we can deem it as an excellent model. For researcher, we always use the AUC of the receiver operating characteristic curve (ROC) to detect a new method’s performance. Usually, we evaluated it by calculating AUC always, whose AUC is higher whose performance is better. Excellent models not only more accurately distinguish between benign pulmonary nodules and malignant pulmonary nodules, but also have higher sensitivity and specificity. In the following sections, we review the four most commonly used as well as widely validated probabilistic models, they are Mayo Clinic, Peking University People’s Hospital (PKUPH), Department of Veterans Affairs (VA) and Brock University. Table 1 summarizes these models’ characteristics [14–18]. The pulmonary nodules benign and malignant four mathematical forecasting model of calculating process has been our detailed generalizations in Table 2.

**Table 1:** Models to estimate the probability of malignancy of patients with pulmonary nodules

Model (year of publication)	Diagnosis method	Number of subjects	Prevalence of malignancy	Nonsmokers included	Nodule size	Statistical methods	Calibration	AUC
Mayo Clinic (1997)	Malignant PNs from a TNAB, bronchoscopy, thoracoscopy, or thoracotomy.	629	23%	Yes	4-30	Logistic regression	Excellent*^,^**	0.833
VA (2007)	Diagnosed by CT、FDG-PET scanning and needle biopsy.	375	54%	Yes	7-30	Logistic regression	Excellent*^,^**	0.790
PKUPH (2012)	Surgical resection and clear pathological diagnosis	371	54%	Yes	9-28	Logistic regression	NR	0.888
Brock (2013)	Diagnosed by histopathological examination or needle-aspiration biopsy.	1871	5.5%	No	1-86	Logistic regression	Excellent**^,^***	0.938

Abbreviations: AUC, area under the curve; CT, computed tomography; FDG-PET, 18F-fluorodeoxyglucose (FDG) positron emission tomography (PET) imaging; LDCT, low-dose computed tomography; NR, not reported; PKUPH, Peking University People’s Hospital; PN, pulmonary nodule; VA, Department of Veterans Affairs.

* The calibration curve plots the observed probability and predicted malignancy probability.

** Calibration refers to the internal verification samples obtained from the model.

*** The calibration is assessed by analyzing the mean absolute error between the observed and predicted malignancy probabilities.

**Table 2:** Probability calculator estimating a pulmonary nodule being lung cancer

Risk variable		Mayo	VA	PU	BU
Demographical	Age(Years)	0.0391	0.0779	0.07	N/A
	Sex(F/M)	N/A	N/A	N/A	0.6467
	Ever smoker(Y/N)	0.7917	2.061	N/A	N/A
	Quit smoke(Years)	N/A	0.0567	N/A	N/A
	Cancer history(Y/N)	1.3388	N/A	N/A	N/A
	Family history of cancer(Y/N)	N/A	N/A	1.267	N/A
Radiological	Upper lobe(Y/N)	0.7838	N/A	N/A	0.6092
	Diameter*(MM)	0.1274	0.112	0.0676	-5.5537*
	Spiculation(Y/N)	1.0407	N/A	0.736	0.9309
	Smooth border(Y/N)	N/A	N/A	-1.408	N/A
	Calcification(Y/N)	N/A	N/A	-1.615	N/A
Model constant		-6.872	− 8.404	-4.496	-6.6144

Abbreviations: BU, Brock University; PU, Peking University; VA, Veteran’s Affairs; F = female, M = male, Y = presence, N = absence

* In the BU model, diameter is defined by (nodule size/10)^−0.5^-1.5811

The resulting number is the *x* in the logistic equation e^*x*^/((1 + e^*x*^)) = risk prediction.

For example, performing the Brock University model prediction for a man and CT examination revealed pulmonary nodules in the lower lobe, without spiculation, and nodules of a size of 10 mm would yield *x* = 0*0.6467 + 0*6092 -[(10/10)^−0.5^-1.5811]*5.5537 + 0*0.9309 − 6.6144 = − 3.3869; plugging into the logistic equation would yield a risk prediction = 0.0327.

## Materials and methods

### Participants

From January 2017 to October 2019, there were 542 patients from Central Hospital of Wuhan with pulmonary nodules who had surgery and had a clear pathological diagnosis. Of the 46 patients who were not included in the study because of incomplete data, we analyzed imaging data from 496 patients. Of the 496 patients with pulmonary nodules, 71 were other lung diseases that were not lung cancer, and 425 were malignant tumors. (Table 3) We usually diagnose lung cancer by examining excised specimens or biopsy specimens histopathologically or cytopathologically. Benign pulmonary nodules need to be stable for more than 2 years and biopsy or surgical resection is no seen in the nodules or a clear diagnosis [19–21].

**Table 3:** Univariate analyses of potential and significant predictors of malignancy

Characteristics	SD	SE	F	95%CI	*P*
Gender (F/M)	0.457	0.021	<0.001	0.26–0.34	0.991
Upper lobe (Y/N)	0.495	0.022	7.883	0.53–0.62	0.387
Family history of cancer (Y/N)	0.288	0.013	0.750	0.07–0.12	0.911
Smoking history (Y/N)	0.492	0.022	8.374	0.36–0.45	0.982
Diameter (mm)	20.656	0.927	<0.001	22.35–25.99	0.028
Spiculation (Y/N)	0.402	0.018	4.880	0.17–0.24	0.290
Calcification (Y/N)	0.500	0.022	1.120	0.43–051	0.673
Ground glass change (Y/N)	0.374	0.017	0.977	0.13–0.20	0.323
Air bronchogram sign (Y/N)	0.219	0.010	0.854	0.03–0.07	0.356
Age (years)	0.005	3.57	25.421	1.068–6.045	<0.001
History of lung cancer (Y/N)	−0.002	0.021	43.099	0.034–0.037	<0.001
Clear border (Y/N)	−0.122	0.042	28.154	−0.203–0.040	<0.001
Lobulation (Y/N)	0.146	0.052	46.852	0.044–0.248	<0.001

Abbreviations: CI, confidence interval; P, significance test; SD, Standard Deviation; SE, Standard Error.

### Variables

All patient information are collected from the hospital information system. Clinical data collected included the patient’s name, serial number, age, sex, history of smoking (smoking years, quit year), history of lung cancer, family history of cancer, nodule characteristics comprised calcification, spiculation, lobulation, clear border, air bronchogram sign, ground glass change, the site of nodules, and nodules diameter. All CT nodule features were collected from the CT reports. The pictures were displayed using lung window setting (width, 1500 HU; level, 600 HU).

### Statistical analysis

In the present study, we employed SPSS21.0 software for statistical analysis. All data sets were included in the single factor analysis to determine the factors affecting the malignant probability of pulmonary nodules. The clinical data of independent and relevant factors related to benign and malignant were screened by multivariate logistic regression. The original prediction performance of the area evaluation model based on (ROC-AUC) with 95% confidence interval is used. *P* value could help us to define whether it has statistically significant or not, when *P* < 0.05 it was normal great. One-way analysis of variance is performed on all observations, and the variance is equal to the conditions of use. If the assumptions are not met then use the Student’s *t*-test [22–25].

## Results

### Synopsis of the characteristics of the model

Compare these four models, we can directly see the Mayo model (patients from the Mayo clinic, 320 men and 309 women), VA model (patients from 10 geographically diverse Veterans Affairs sites in the United States, 367 men, 8 women), PKUPH model (patients from Peking University People’s Hospital in china, 197 men and 174 women), Brock university model (patients from the Pan-Canadian Early Detection of Lung Cancer Study, 985 men, 886 women). Depending on the population, the characteristics of the four models as predictors also show their differences. The Mayo Clinic model focuses on predictive features such as age, smoking, nodule diameter, spiculation, over 5 years related extrathoracic cancer, and upper lobe location. VA model includes smoking, age, nodule diameter and quit time as features. PKUPH model comprises age, nodule diameter, calcification, spiculation, edges and family history of cancer as factors. Brock university model incorporates gender, nodule size, upper lobe location, spiculation as features.

### A comparison of the four models

**Figure 1:**
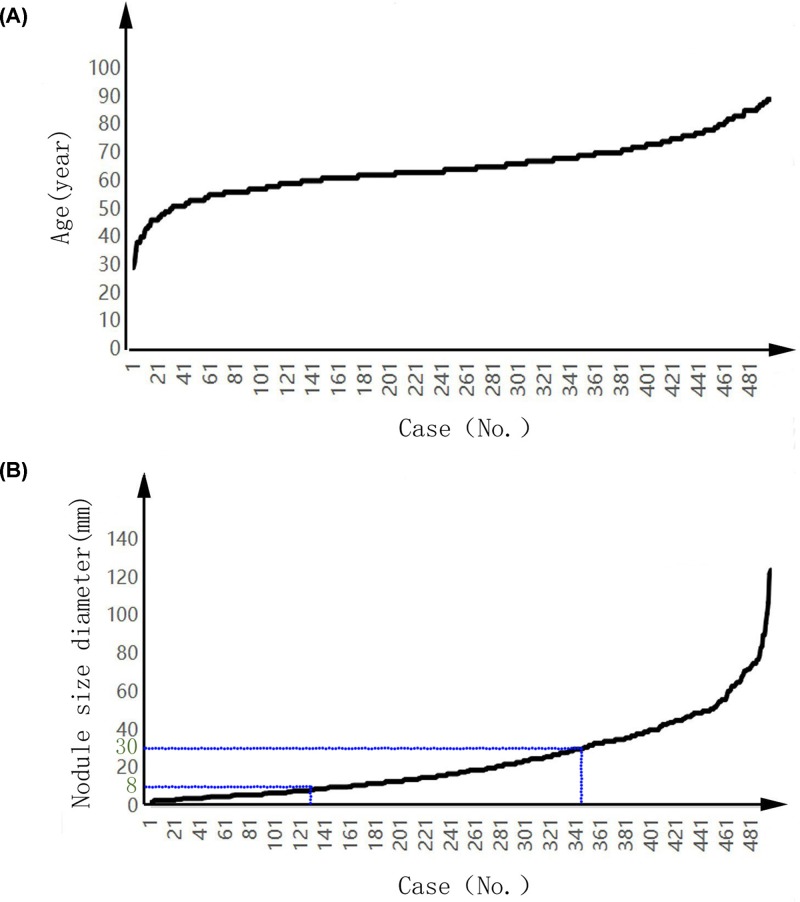
Patient information visual map (**A**) Age distribution of 496 patients. (**B**) Distribution of pulmonary nodules in 496 patients.

**Figure 2:**
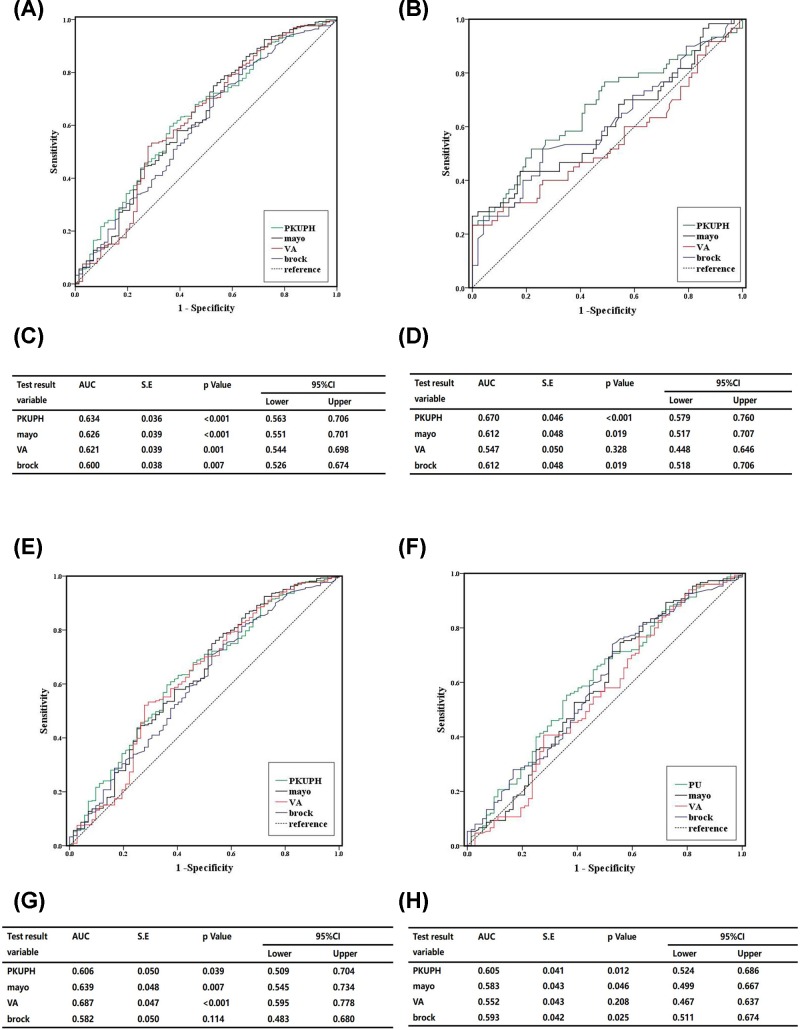
A comparison and evaluation of the four models (**A** and **C**) A comparison of the four models, the value of AUC of PKUPH model is 0.634, the value of AUC of Mayo model is 0.626, the value of AUC of VA model is 0.621, and the value of AUC of Brock model is 0.600. (**B** and **D**) A comparison of the third and fourth phases of the first and second phases of lung cancer. The value of AUC for PKUPH model is 0.670, the value of AUC for Mayo model is 0.621, the value of AUC for VA model is 0.547, and the value of AUC for Brock model is 0.612. (**E** and** G**) A comparison of lung squamous cell carcinoma, the value of AUC of PKUPH model was 0.606, the value of AUC of Mayo model was 0.639, the value of AUC of VA model was 0.687, and the value of AUC of Brock model was 0.582. (**F** and **H**) A comparison of lung adenocarcinoma, the value of AUC of PKUPH model was 0.605, the value of AUC of Mayo model was 0.583, the value of AUC of VA model was 0.552, and the value of AUC of Brock model was 0.593.

On account of Brock university’s model that is excellent in all aspects, we expected it to perform well, but by comparing the diagnostic efficiency of the four models, we found that PKUPH model is more suitable for our patients. In the data that we collected, during this period, 46 participators (8.45%) were lost to follow-up, and of the 425 patients (299 men, 126 women) with malignant pulmonary nodules, 150 had lung adenocarcinoma and 56 had lung squamous cell carcinoma. The patient’s age ranged from 29 to 89 years (Figure 1A). In addition, there were 126 patients with pulmonary nodules between 1.9 and 8 mm in diameter, 219 patients with pulmonary nodules between 8 and 30 mm in diameter, and 151 patients with pulmonary nodules between 30 and 124 mm in diameter. (Figure 1B). We bring the information of these collected patients into the model’s formula and calculate the results. We performed logistic regression on the results obtained and compared their AUC. In the comparison of the four models, the value of AUC of PKUPH model is 0.634, the value of AUC of Mayo model is 0.626, the value of AUC of VA model is 0.621, and the value of AUC of Brock model is 0.600. (Figure 2A,C). In the comparison of the third and fourth phases of the first and second phases of lung cancer. The value of AUC for PKUPH model is 0.670, the value of AUC for Mayo model is 0.621, the value of AUC for VA model is 0.547, and the value of AUC for Brock model is 0.612. (Figure 2B,D). In the comparison of lung squamous cell carcinoma, the value of AUC of PKUPH model was 0.606, the value of AUC of Mayo model was 0.639, the value of AUC of VA model was 0.687, and the value of AUC of Brock model was 0.582. (Figure 2E,G). When it comes to lung adenocarcinoma, the value of AUC of PKUPH model was 0.605, the value of AUC of Mayo model was 0.583, the value of AUC of VA model was 0.552, and the value of AUC of Brock model was 0.593. (Figure 2F,H).

### Evaluation of suitability

In the comparison of these models, we found that PKUPH model May show relatively better. It includes the diagnostic efficiency of lung cancer and pulmonary nodules, lung adenocarcinoma and early and late lung cancer. But in the comparison of squamous cell carcinoma, the VA model will be more suitable. In the supplementary material, we also provide a detailed table of logistic regression for the four models, (Tables 4,5,6 and 7) and four kinds of logistic regression model to compare (Table 8).

**Table 4:** Mayo Clinic Model Logistic Regression

	*B*	S.E	Wals	df	*P*	OR	95%CI	
							Lower	Upper
Gender	0.761	0.738	1.063	1	0.303	2.140	0.504	9.087
Age	0.022	0.031	0.478	1	0.489	1.022	0.961	1.087
Upper	0.213	0.549	0.151	1	0.698	1.237	0.422	3.629
Family	2.947	1.920	2.357	1	0.125	19.051	0.442	820.388
History	-18.432	7479.852	\	1	0.998	\	\	\
Smoke	1.081	0.696	2.413	1	0.120	2.949	0.754	11.537
Diameter	-0.002	0.013	0.013	1	0.908	0.998	0.972	1.025
Spiculation	0.154	0.706	0.048	1	0.827	1.167	0.292	4.656
Calcification	0.521	0.578	0.812	1	0.368	1,684	0.542	5.231
Clear border	1.486	0.727	4.172	1	0.041	4.418	1.062	18.384
Upper lobe	-0.656	0.661	0.984	1	0.321	0.519	0.142	1.897
CTR	-1.468	1.139	1.660	1	0.198	0.230	0.025	2.148
Quitsmoke	0.649	0.833	0.608	1	0.436	1.914	0.374	9.791

Abbreviations: B, degree of freedom; CI, confidence interval; df, degree of freedom; OR, odds ratio; *P*, significance test; S.E., Standard Error; Wals, Chi-square value.

**Table 5:** Department of Veterans Affairs Model Logistic Regression

	*B*	S.E	Wals	df	*P*	OR	95%CI	
							Lower	Upper
Gender	2.123	0.837	6.429	1	0.011	8.360	1.619	43.158
Age	-0.006	0.033	0.038	1	0.846	0.994	0.932	1.060
Upper	-0.114	0.571	0.040	1	0.842	0.892	0.291	2.733
Family	18.786	5750.353	\	1	0.997	\	\	\
History	-18.178	10381.213	\	1	0.999	\	\	\
Smoke	2.195	0.872	6.336	1	0.012	8.981	1.626	49.618
Diameter	-0.012	0.011	1.017	1	0.313	0.989	0.967	1.011
Spiculation	0.481	0.777	0.383	1	0.536	1.617	0.352	7.421
Calcification	0.627	0.646	0.944	1	0.331	1.872	0.528	6.638
Clear border	1.750	0.732	5.718	1	0.017	5.757	1.371	24.173
Upper lobe	-1.302	0.868	2.253	1	0.133	0.272	0.050	1.489
CTR	-32.974	8132.225	\	1	0.997	\	\	\
Quitsmoke	0.338	0.715	0.223	1	0.637	1.402	0.345	5.696

Abbreviations: B, degree of freedom; CI, confidence interval; df, degree of freedom; OR, odds ratio; *P*, significance test; S.E., Standard Error; Wals, Chi-square value.

**Table 6:** Peking University People’s Hospital Model Logistic Regression

	*B*	S.E	Wals	df	*P*	OR	95%CI	
							Lower	Upper
Gender	0.896	0.491	3.334	1	0.068	2.451	0.936	6.414
Age	0.017	0.024	0.508	1	0.476	1.017	0.971	1.065
Upper	0.303	0.376	0.648	1	0.421	1.353	0.648	2.828
Family	0.492	0.881	0.312	1	0.577	1.636	0.291	9.203
History	-19.092	6511.017	\	1	0.998	\	\	\
Smoke	0.599	0.492	1.480	1	0.224	1.820	0.694	4.774
Diameter	0.014	0.010	1.929	1	0.165	1.014	0.994	1.035
Spiculation	0.282	0.452	0.389	1	0.533	1.326	0.547	3.215
Calcification	0.712	0.465	2.352	1	0.125	2.039	0.820	5.068
Clear border	2.034	0.698	8.487	1	0.004	7.645	1.946	30.038
Upper lobe	-0.760	0.525	2.101	1	0.147	0.467	0.167	1.307
CTR	-0.341	0.503	0.460	1	0.497	0.711	0.265	1.906
Quitsmoke	-0.063	0.414	0.023	1	0.878	0.939	0.417	2.113

Abbreviations: B, degree of freedom; CI, confidence interval; df, degree of freedom; OR, odds ratio; *P*, significance test; S.E., Standard Error; Wals, Chi-square value.

**Table 7:** Brock University Model Logistic Regression

	*B*	S.E	Wals	df	*P*	OR	95%CI	
							Lower	Upper
Gender	0.646	0.724	0.796	1	0.372	1.908	0.461	7.890
Age	-0.005	0.032	0.026	1	0.871	0.995	0.935	1.058
Upper	0.330	0.639	0.267	1	0.605	1.392	0.398	4.871
Family	2.599	1.782	2.127	1	0.145	13.454	0.409	442.574
History	-18.806	10974.389	\	1	0.999	\	\	\
Smoke	0.872	0.738	1.395	1	0.238	2.392	0.563	10.166
Diameter	0.004	0.014	0.093	1	0.761	1.004	0.976	1.033
Spiculation	-0.225	0.715	0.100	1	0.752	0.798	0.197	3.238
Calcification	0.095	0.600	0.025	1	0.874	1.100	0.339	3.565
Clear border	0.831	0.801	1.075	1	0.300	2.295	0.477	11.034
Upper lobe	-0.944	0.733	1.660	1	0.198	0.389	0.093	1.636
CTR	-0.468	0.842	0.309	1	0.578	0.626	0.120	3.259
Quitsmoke	-0.096	0.796	0.015	1	0.904	0.908	0.191	4.321

Abbreviations: B, degree of freedom; CI, confidence interval; df, degree of freedom; OR, odds ratio; *P*, significance test; S.E., Standard Error; Wals, Chi-square value.

**Table 8:** Comparison of Models’ Logistic Regression

	*B*	S.E	Wals	df	*P*	OR	95%CI	
							Lower	Upper
PKUPH	1.875	0.703	7.110	1	0.008	6.524	1.644	25.896
Mayo	0.284	1.364	0.043	1	0.835	1.328	0.092	19.240
VA	0.546	0.876	0.388	1	0.533	1.726	0.310	9.618
Brock	-1.219	1.369	0.792	1	0.374	0.296	0.020	4.329
Constant	0.637	0.301	4.479	1	0.034	1.891		

Abbreviations: B, degree of freedom; CI, confidence interval; df, degree of freedom; OR, odds ratio; *P*, significance test; S.E., Standard Error; Wals, Chi-square value.

## Discussion

Selection of mathematical prediction models for pulmonary nodules requires caution, radiologists should consider in their area of lung cancer epidemiology, and verify it in the local population. It has brought many problems to the clinic, since there are many factors affecting the benign and malignant lung nodules. Different prediction models use different predictive factors, which greatly affects the applicability of the model. These models have their own advantages, many studies have clarified them. At the same time, they also have some problems to be considered, and their applicability in different region needs to be supported by more data. For example, the Mayo model is based on chest X-rays, but for more sensitive and accurate screening of lung nodules, people now use CT as a screening method. At the same time, the Mayo model is not suitable for people who have been diagnosed with cancer or Patients with a history of lung cancer. The VA model does not contain radiological characteristics and is not suitable for nodules smaller than 7 mm. The PKUPH Model excludes patients with intrapulmonary and extrapulmonary malignancies within 5 years. The Brock Model is suitable neither for screening low-risk populations, nor for patients with hilar or mediastinal lymphadenopathy. By comparing AUC, we found that the PKUPH Model is more suitable for patients with PNs in the region, even if it was not specifically calibrated. The AUC value of the Mayo model is second only to that of the PKUPH model. The VA model is more suitable for identifying patients with lung squamous cell carcinoma, while the Brock model and the PKUPH model are more suitable for identifying patients with lung adenocarcinoma. The reason for this result is because of different nationalities (the prevalence of tobacco and difference in the pattern of the history of tobacco exposure) conditions together with geographic disparities differences. Most lung cancers (61%) were diagnosed as stage III or IV; Only 21% of cases have been confirmed in stage I. As for stage I 5-year survival rates were 57%, while stage IV was 4%. Almost 75% of lung cancer survivors are 65 years old and above, more than 60% of patients diagnosed in five years, it is due to the low survival of lung cancer [26–31]. In the 2018 Cancer Statistics, invasive cancer men (39.7%) and women (37.6%). In the 2019 Cancer statistics, invasive cancer men (39.3%) and women (37.7%). It reflects to some extent the cause of the differences in environmental exposure [28,30]. So far, the cancer of lung cancer is the biggest geographical differences, reflecting the state between smoking prevalence of huge differences in history and continued. It can be also seen that in the United States, the occurrence of lung cancer in men is decreasing (estimate the amount of new lung cancer cases at 12,1680 in 2018 and 11,6440 in 2019), while the incidence of women is rising (estimate the amount of new lung cancer cases at 11,350 in 2018 and 11,710 in 2019). It’s worth noting that there is no significant difference between females from Chinese (22.8 per 100,000) and some Western European countries, (for instance, in France 22.5 per 100,000) although there are real differences in smoking prevalence between the two types of people. The incidence and trend of lung cancer vary greatly depending on gender, age, ethnicity, and socioeconomic status. In the United States, lung cancer mortality is highest among men of lower socioeconomic status, especially in central and southern regions. Smoking rates are decreasing globally, especially among men, such as the United States, the United Kingdom, and Australia. But in countries that started smoking late, we are seeing an increase in smoking rates. Nowadays, there is more than 50% lung cancer patients died every year in low income countries as well as middle income ones. [4,5,28,30].

Studies have shown that exposure to central bronchi by low molecular weight polycyclic aromatic hydrocarbons produced by smoking can lead to small cell lung cancer, while nitrosamines in peripheral lung tissue exposed to tobacco smoke can cause lung adenocarcinoma. All histological types were closely related to smoking, the relative risk of adenocarcinoma is much lower than that of small cell lung cancer and squamous cell carcinoma. The former of which is the more common of non-smokers and women, while the latter of which are more common with the time of smoke increased. In general, lung cancer in non-smokers are different from that in smokers at the molecular as well as epigenetic levels. Histologically, cancer from never-smokers is also different from smoker patients’ cancer. Never smokers and women are mainly influenced by lung adenocarcinoma, while male smokers are predominantly squamous cell carcinoma [32–35].

There is increasing evidence to suggest recommendations to manage these patients, including how to define risk for progression and how to how to analyze who can observe through continuous imaging. These imaging features also assist to distinguish patients who may have early stage lung cancer that profit from local treatment. But this is not enough, and this may require the creation of new models to meet the needs of patients and clinicians with lung nodules in the region. Because the model is also constantly improving, by which kinds of indicators are included in the calculation. Obtaining a more accurate formula is a difficult problem. At the same time, we found that it is not rigorous to consider only smoking, but the time and amount of smoking. There are many factors influencing. Different places may have different results due to different geographical environments, different living habits, and different eating habits. This requires researchers to consider and verify whether other models can be directly used. Looking through the literature reports in recent years, it is not difficult to find that more and more better models have been established. More indicators or more sensitive molecular markers may be added (e.g. CEA and Cyfra21-1). It is complex and challenging to evaluate lung nodules. At present, guidelines advocate a scheme of a system based on clinical and radiographic features to evaluate the likelihood of malignancy. An externally validated clinical malignancy probabilistic model can assist us to identify benign and malignant nodules and advise clinicians and patients in making management decisions. As we apply the model to the clinic, it is significant to know the source population of each model. Therefore, it is very important to establish a regional prediction model for the benign and malignant pulmonary nodules, which might possess potential to help doctors to choose and interpret diagnostic and reduce the cost and suffering of patients. The development of radionomics and molecular biomarkers is expected to enhance the probability estimation of malignant tumors in the near future.

## Data Availability

Data sets mentioned in this survey (including normal patient data sets and lost follow-up data sets) can be obtained from the corresponding authors as long as reasonably required.

## Abbreviations

AUC: area under the curve
CI: confidence interval
CT: computed tomography
LDCT: low-dose computed tomography
PET: positron emission tomography
